# Chronic lung infection by *Pseudomonas aeruginosa* biofilm is cured by L-Methionine in combination with antibiotic therapy

**DOI:** 10.1038/srep16043

**Published:** 2015-11-02

**Authors:** Divya Prakash Gnanadhas, Monalisha Elango, Akshay Datey, Dipshikha Chakravortty

**Affiliations:** 1 Department of Microbiology and Cell Biology, Indian Institute of Science, Bangalore, India.; 2 Department of Aerospace Engineering, Indian Institute of Science, Bangalore, India.; 3 The Bioengineering Program, Indian Institute of Science, Bangalore, India

## Abstract

Bacterial biofilms are associated with 80–90% of infections. Within the biofilm, bacteria are refractile to antibiotics, requiring concentrations >1,000 times the minimum inhibitory concentration. Proteins, carbohydrates and DNA are the major components of biofilm matrix. *Pseudomonas aeruginosa* (PA) biofilms, which are majorly associated with chronic lung infection, contain extracellular DNA (eDNA) as a major component. Herein, we report for the first time that L-Methionine (L-Met) at 0.5 μM inhibits *Pseudomonas aeruginosa* (PA) biofilm formation and disassembles established PA biofilm by inducing *DNase* expression. Four *DNase* genes (*sbcB*, *endA*, *eddB* and *recJ*) were highly up-regulated upon L-Met treatment along with increased DNase activity in the culture supernatant. Since eDNA plays a major role in establishing and maintaining the PA biofilm, DNase activity is effective in disrupting the biofilm. Upon treatment with L-Met, the otherwise recalcitrant PA biofilm now shows susceptibility to ciprofloxacin. This was reflected *in vivo*, in the murine chronic PA lung infection model. Mice treated with L-Met responded better to antibiotic treatment, leading to enhanced survival as compared to mice treated with ciprofloxacin alone. These results clearly demonstrate that L-Met can be used along with antibiotic as an effective therapeutic against chronic PA biofilm infection.

Most of the microorganisms form micro-colonies and produce extracellular matrix to form biofilm. Biofilm formation is a lifestyle commonly adopted by a variety of microorganisms whether in the environment or in clinical settings. These biofilms are formed upon adherence to a living or non-living surface, formation of micro-colonies and finally the production of a resilient extracellular matrix. While the composition of this extracellular matrix might vary slightly in different microbes, biofilms endow the bacteria a unique resistance against antibiotics and other anti-microbial agents. This reduced susceptibility may be due to the presence of extracellular polymer matrix which acts as a physical barrier to diffusion (intrinsic) or due to the transfer of extrachromosomal DNA from resistant organisms to susceptible organisms (acquired)^1^. These characteristics make the clinical management of these biofilm infections very difficult.

*Pseudomonas aeruginosa* (PA), a Gram-negative bacterium can form biofilms on a variety of surfaces such as lungs, contact lenses and contaminated catheters^2^,^3^. The formation of PA biofilm is initiated by the attachment of free bacteria to a surface, followed by formation of micro-colonies. Once the micro-colony matures as a biofilm, free bacteria exit from the biofilm structure to occupy a new surface^4^. PA biofilms, while poorly defined, are known to contain a mix of polysaccharides, nucleic acids and proteins in their matrix or EPS^5^,^6^,^7^,^8^. Extracellular DNA (eDNA) is an important component of the PA biofilm matrix^5^,^6^,^7^,^8^. The eDNA, derived from random chromosomal DNA, is required for the biofilm formation, integrity and motility^5^,^6^,^7^,^8^,^9^,^10^. It has been observed that eDNA is required for initial bacterial adhesion, cellular aggregation as well as biofilm strength^11^,^12^,^13^. Further, it protects the bacteria against antibiotics and detergents^10^,^13^. The source of eDNA is not completely understood and autolysis during microcolony formation may be one of the mechanisms underlying its production^14^. In PA, quorum sensing (QS) dependent mechanisms, which involve N-acyl-L-homoserine lactones (AHL) and the *Pseudomonas* quinolone signaling (PQS) molecule, are known to regulate eDNA release. QS independent mechanism of eDNA secretion via type IV pili and flagella has also been demonstrated^5^,^14^.

PA, an opportunistic human pathogen, can cause life-threatening infections in cystic fibrosis (CF) patients and individuals with a compromised immune system^15^,^16^. Since antibiotic concentrations 1,000 times more than MIC are required to kill the bacteria embedded within the biofilm matrix, avenues for biofilm disassembly are a topic of active research. Corosolicacid and asiatic acid^17^, 3-indolylacetonitrile^18^, brominated furanones^19^, garlic^20^, ursine triterpenes^21^, and ginseng^22^ are some of the compounds which have anti-biofilm activity. However, the mechanism of these anti-biofilm activities is not completely understood.

Apart from these molecules, D-amino acids (D-Leu, D-Met, D-Trp and D-Tyr), produced at the late phase of biofilm development, have been shown to trigger the disassembly of the existing biofilms of *Bacillus subtilis* and *Staphylococcus aureus*^23^,^24^. Apart from D-amino acids, L-Tryptophan is also known to inhibit the formation of PA biofilms and partially disassemble the established biofilms at 10 mM concentration^25^. In the present work, we found that only the L-isoform and not the D-isoform of Methionine (Met) inhibited PA biofilm formation and disassembled the existing mature biofilm. Treatment with L-Met was associated with a concomitant increase in the DNase activity in the biofilm culture supernatant and four DNase genes were highly up-regulated at mRNA level. Our results suggest that L-Met induced *DNase* secretion and eDNA degradation might be the mechanism underlying biofilm inhibition and disassembly. In a mouse model of chronic PA lung infection, L-Met treatment in combination with antibiotics could rescue the mice whereas antibiotic therapy alone was ineffective. To our knowledge, this is the first report demonstrating the therapeutic potential of L-Met through its inhibitory effect on the formation and maintenance of PA biofilm.

## Results

### L-Methionine (L-Met) inhibits PA biofilm formation

**Figure 1:**
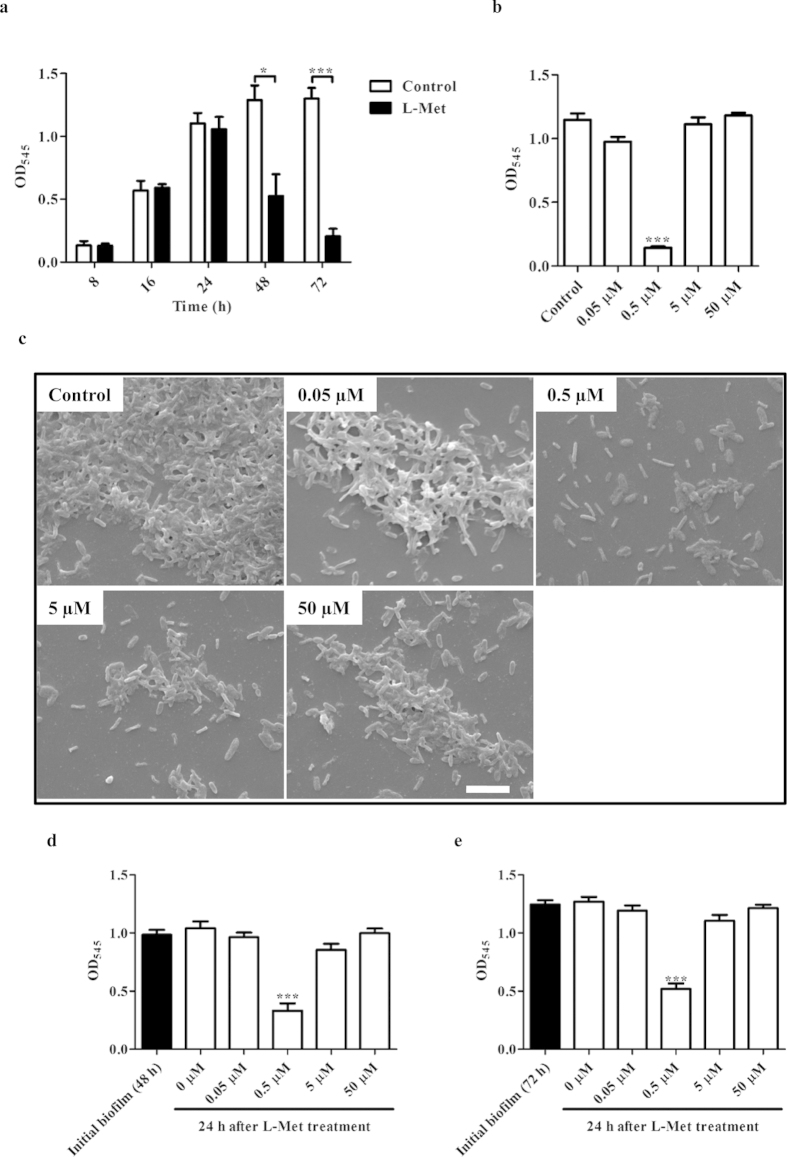
L-Methionine (L-Met) inhibits and disassembles PA biofilm. **(a)** 24-well plate containing PA biofilm was confirmed by crystal violet staining. At different duration of incubation with or without L-Met (0.5 μM) at static condition, the culture was removed and crystal violet staining was performed and optical density was determined at 545 nm (OD_545_). **(b)** PA was incubated with different concentrations of L-Met and at 72 h, the biomass was determined by crystal violet staining. (0.5 μM was compared with control) **(c)** A coverslip was placed in a 24-well plate and 72 h old biofilm was observed with Scanning electron microscopy. Scale bar: 10 μM. **(d)** 48 h old PA biofilm and **(e)** 72 h old PA biofilm were incubated with different concentrations of L-Met and the biomass was determined by crystal violet staining. (0.5 μM was compared with 0 μM). Statistical significance was calculated using One-way ANOVA. Asterisks indicate statistical significance as follows: *(p < 0.05), ***(p < 0.001).

Effect of exogenous addition of L/D-amino acids, at different concentrations, on the growth of PA biofilms was tested, of which L-Methionine (L-Met) was found to inhibit PA biofilm at 0.5 μM concentration at 72 h. PA biomass reduced by 75% when incubated with 0.5 μM L-Met for 72 h (Fig. 1a). Interestingly, this effect on biofilm biomass, was specific only to the 0.5 uM concentration of L-Met and was not observed at higher concentrations (Fig. 1b,c). Growth of PA was unaffected in the presence of 0.5 μM L-Met in Luria broth (Fig. S1a) as well as M9 minimal media (Fig. S1b) under shaking condition (180  rpm) at 37 °C suggesting that the decrease in biofilm formation was not a result of growth inhibition by L-Met. Furthermore, only L-Met and not D-Met was able to inhibit PA biofilm (Fig. S2). The inhibitory effect of mM concentrations of tryptophan on PA biofilm formation was also observed in our study, however a similar effect was exerted by L-Met at a much lower concentration^25^ (Fig. 1b).

### L-Met disassembles established PA biofilms

Next we examined whether L-Met could disassemble the established PA biofilms. CV staining revealed that L-Met treatment could disassemble the established PA biofilms (Fig. 1c,d). Initial biofilm formation was determined at 48 h or 72 h of incubation. The medium was then removed and replaced with fresh LB medium with various concentrations of L-Met (0.0 to 50.0 μM). The plate was incubated for an additional 24 h at 25 °C. Treatment with 0.5 μM L-Met resulted in disassembly of 48 h old biofilms (Fig. 1c) as well as 72 h old biofilms (Fig. 1d). Biofilm treatments with lower concentration of L-Met (<0.5 μM) or higher concentration of L-Met (>5 μM) prevented further biofilm growth but did not cause any disassembly of the established biofilm.

### L-Met degrades extracellular DNA (eDNA) by inducing *DNase* expression in PA

**Figure 2:**
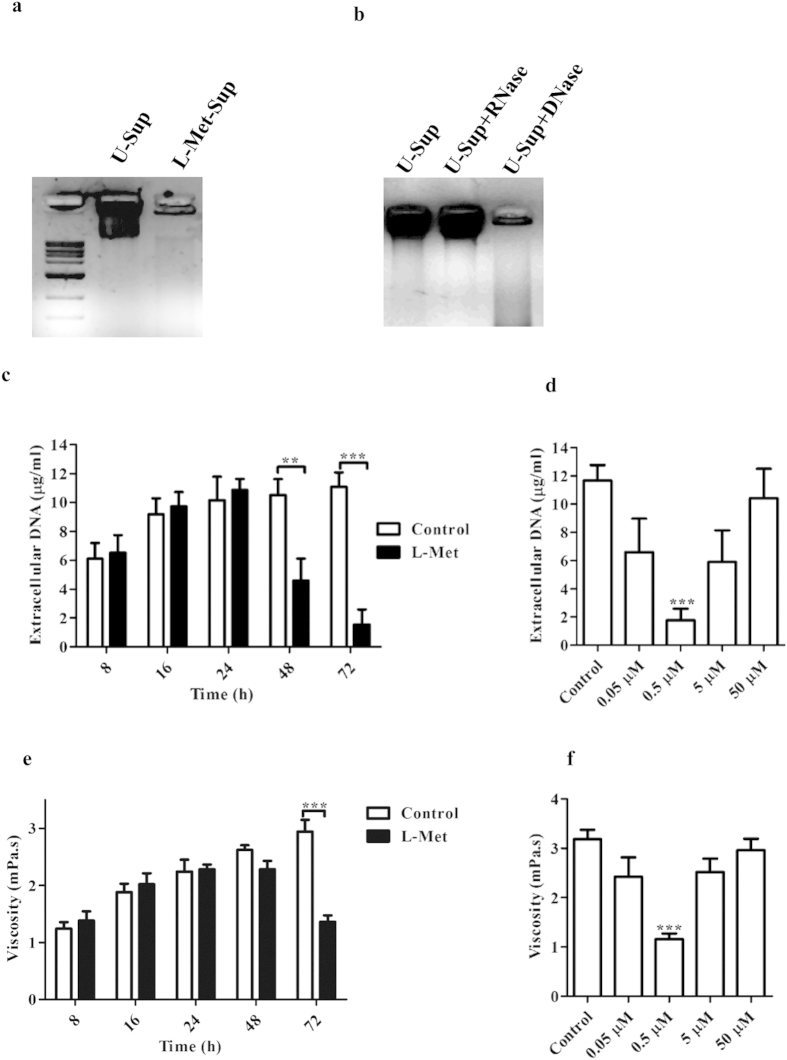
L-Methionine (L-Met) degrades extracellular DNA. 72 h old PA biofilm culture media was centrifuged at 2000 g for 5 min and the supernatant of **(a)** Untreated culture (U-Sup) & **(b)** L-Met treated culture (L-Met-Sup) were loaded in the agarose gel and visualized. **(b)** U-Sup was treated with DNase and RNase (Fermentas) for 1 h at 37 °C and then loaded in the agarose gel and visualized. Extracellular DNA was precipitated and DNA concentration was determined by spectrofluorimetrically from U-Sup and L-Met-Sup **(c)** with different duration of incubation with L-Met (0.5 μM) or **(d)** with different concentrations of L-Met and incubated for 72 h. Viscosity of U-Sup and L-Met-Sup **(e)** with different duration of incubation with L-Met (0.5 μM) or **(f)** with different concentrations of L-Met and incubated for 72 h was determined using Rheometer. (D & F - 0.5 μM was compared with control). Statistical significance was calculated using One-way ANOVA. Asterisks indicate statistical significance as follows: **(p < 0.005), ***(p < 0.001).

Formation of PA biofilm, seen after 24 h of incubation, was accompanied by a marked increase in the viscosity of the medium. This viscosity was visibly absent upon treatment with L-Met. Earlier reports have shown that PA biofilms have extracellular DNA as a major component. We hypothesized that the observed decrease in PA biofilm upon treatment was due to a reduction in the eDNA component of the matrix. 72 h old PA biofilm culture supernatant was loaded in a 1.0% agarose gel and it was visible that L-Met treated PA biofilm culture media (L-Met-Sup) lacked DNA whereas, high concentration of DNA was present in the untreated PA culture media (U-Sup) (Fig. 2a). This DNA was sensitive to degradation by exogenous DNase, but was unaffected by RNase treatment as expected (Fig. 2b). PA was incubated at static condition at 25 °C and at different time points eDNA was extracted from the culture media and quantified spectrofluorimetrically^26^. The eDNA was present in the PA biofilm culture medium up to 24 h. However at 48 h and 72 h the amount of eDNA was significantly reduced (Fig. 2c). Four-fold reduction in the eDNA was observed in L-Met treated culture medium compared to the control. Treatment with specifically 0.5 μM L-Met reduced eDNA in a 72 h old PA biofilm culture media and no significant reduction in the eDNA concentration was observed with higher or lower concentration of L-Met (Fig. 2d). These results corroborate with the changes in the viscosity of PA biofilm culture media at different time period of after L-Met addition (Fig. 2e) and at different concentration of L-Met after 72 h of incubation (Fig. 2f).

**Figure 3:**
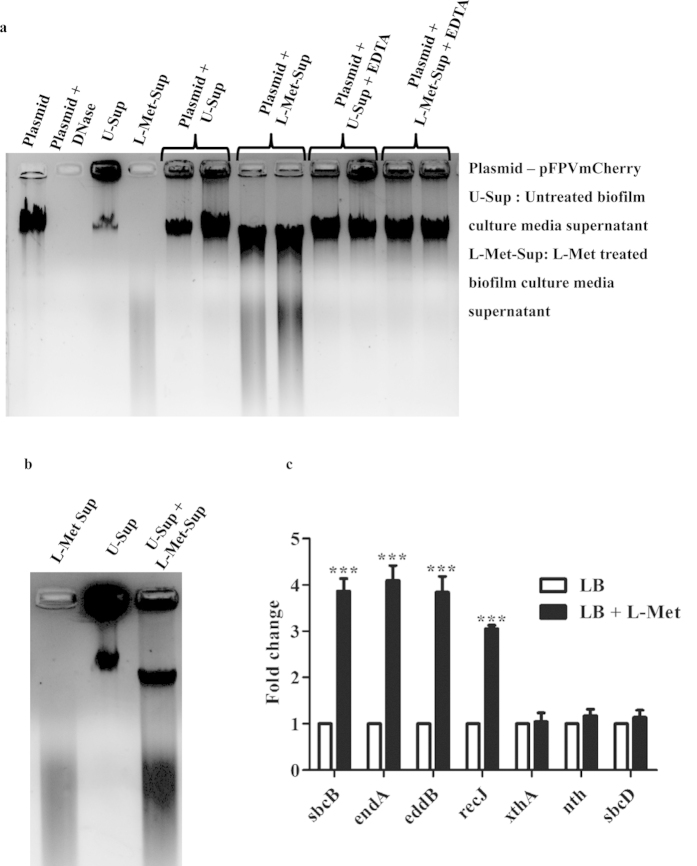
DNase activity. **(a)** 100 μl of 72 h old U-Sup or L-Met-Sup were incubated with equal volume of pFPV/mcherry (1 μg/μl) with or without 0.1% EDTA for 1h at 37 °C and the samples were loaded in 1% agarose gel and visualized. **(b)** 100 μl of U-Sup and L-Met-Sup were mixed and incubated for 1 h at 37 °C, loaded in 1% agarose gel and visualized. **(c)** QPCR analysis of the expression of different *DNase* genes with or without L-Met treatment. Expression was normalized to the housekeeping gene *16s rRNA.*The Statistical significance was calculated using One-way ANOVA. Asterisks indicate statistical significance as follows: ***(p < 0.001)

Studies have shown that eDNA, while being a structural component of the PA biofilm, can also serve as an alternative nutrient source. Upon biofilm maturation and in nutrient limiting conditions, the bacteria are known to secrete DNase (EddB/PA3909) which aids in eDNA degradation which is used for bacterial growth and eventual dispersal^27^. We hypothesized that the biofilm inhibition observed might correlated with a change in the DNase activity in the biofilm. In order to examine this, the biofilm culture media [Untreated (U-Sup) or L-Met treated (L-Met-Sup)] was centrifuged at 2000 g for 5 min and the supernatant was incubated with pFPV/mcherry plasmid DNA. No visible plasmid degradation was observed when the plasmid was incubated with U-Sup whereas, plasmid degradation was observed when incubated with L-Met-Sup (Fig. 3a). Plasmid degradation was abrogated upon addition of EDTA to the L-Met-Sup (Fig. 3a). This suggests that the L-Met-Sup has an increased DNase activity. To further confirm this, L-Met-Sup was mixed with U-Sup for 1 h and the mixture was run in a 1.0% agarose gel. A clear reduction in the DNA quantity was observed near the well along with a smear in the gel (Fig. 3b). These results clearly indicate the presence of heightened DNase activity in the L-Met-Sup at 72 h.

In order to gain an insight into the regulation of DNase activity in the L-Met-Sup, QPCR was performed for different *DNase* genes in PA (Table 1). In L-Met treated 72 h old PA biofilm, *sbcB* (Exodeoxyribonuclease I), *endA* (DNA-specific endonuclease I), *eddB* or PA3909 (Extracelullar DNA degradation protein, EddB) and *recJ* (Single-stranded-DNA-specific exonuclease, RecJ) showed 3 to 4 fold higher expression compared to the untreated PA biofilm (Fig. 3c). The other *DNase* genes including *xthA* (Exodeoxyribonuclease III), *nth* (Endonuclease III) and *sbcD* (Exonuclease SbcD) showed no difference. This suggests that L-Met treatment results in transcriptional up-regulation of four of the *DNase* genes were in PA biofilm. Sub-cellular localization of these DNases was predicted using an online prediction tool, PredictProtein^28^. Apart from EddB, a secreted DNase^27^, all other DNases were predicted as cytoplasmic (Table 2). This up-regulated eddB may play an important role in eDNA degradation and hence inhibition of the biofilm.

### L-Met inhibits PA motility

**Figure 4:**
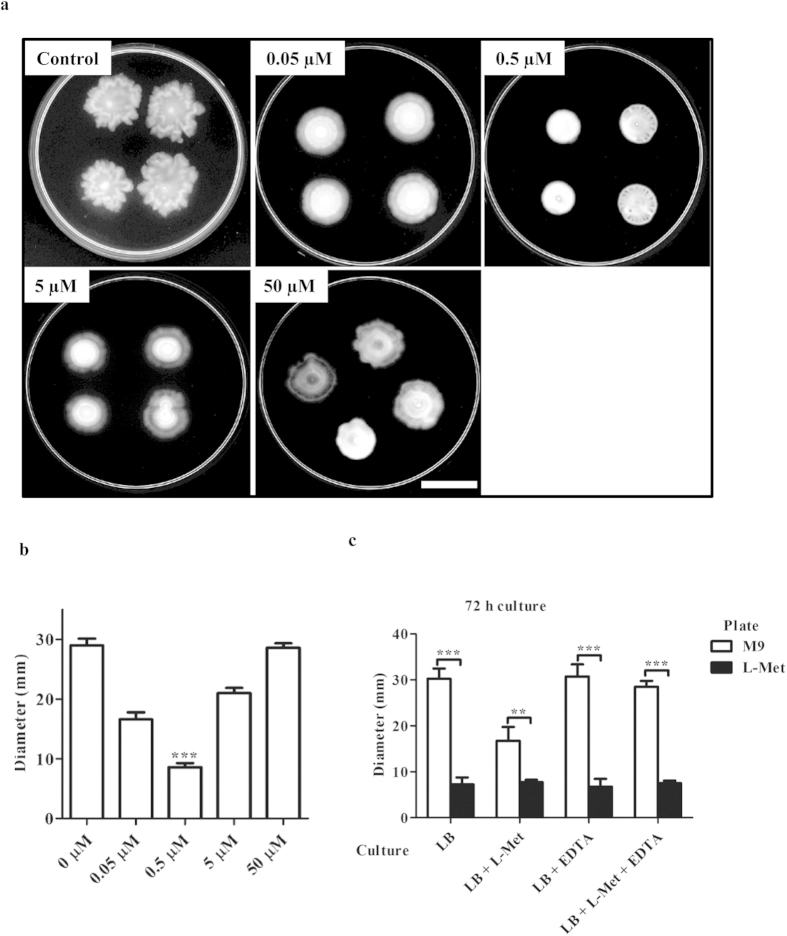
L-Met inhibits *Pseudomonas aeruginosa* motility. **(a)** Stationary PA was spotted on M9 agar plates containing various concentrations of L-Methionine (0.05 μM, 0.5 μM, 5 μM & 50 μM). The plates were incubated at 30 °C for 24 h and photograph was taken. Scale bar: 2 cm **(b)** The colony diameter was measured and plotted. (0.5 μM was compared with 0 μM) **(c)** 72 h old PA biofilm culture grown in LB, LB+L-Met (0.5 μM), LB+EDTA (0.1%), LB+L-Met+EDTA was inoculated in M9 agar plates with or without L-Met (0.5 μM). After 24 h of incubation at 30°C, the colony diameter was measured and plotted.The Statistical significance was calculated using One-way ANOVA. Asterisks indicate statistical significance as follows: **(p < 0.01), ***(p < 0.001).

It has been observed that bacterial motility and biofilm formation have a close relationship^29^,^30^,^31^, and hence we investigated L-Met’s effect on PA motility. Swarming motility was assessed in 0.5% M9 agar plate^32^ with different concentrations of L-Met (0.0 to 50.0 μM). When 5 μl (1 × 10^4^) overnight PA culture was added to 0.5% M9 agar plate with different concentrations of L-Met, swarming motility was decreased by approximately 30% at 0.5 μM L-Met (Fig. 4a,b). In contrast, higher concentration (50 μM) of L-Met did not show any significant decrease in the motility. PA was incubated in LB, LB+L-Met, LB+EDTA or LB+L-Met+EDTA for 72 h and 5 μl of the culture was added to 0.5% M9 agar or 0.5% M9 agar with 0.5 μM L-Met. PA culture grown in the presence of L-Met showed decreased motility (Fig. 4c) however; incubation with L-Met+EDTA reversed the motility defect as seen on the M9agar without L-Met (Fig. 4c). All the culture showed decreased motility when they are inoculated in M9 agar with 0.5 μM L-Met (Fig. 4c). Similarly, a reduction in the twitching motility was also observed (Fig. S3). It is known that eDNA plays a major role in the motility and they are required for the establishment of the biofilm^9^. These results indicate that the eDNA was affected in the presence of L-Met resulting in diminished bacterial the motility.

### L-Met increases antibiotic sensitivity of PA biofilm *in vitro*

**Figure 5:**
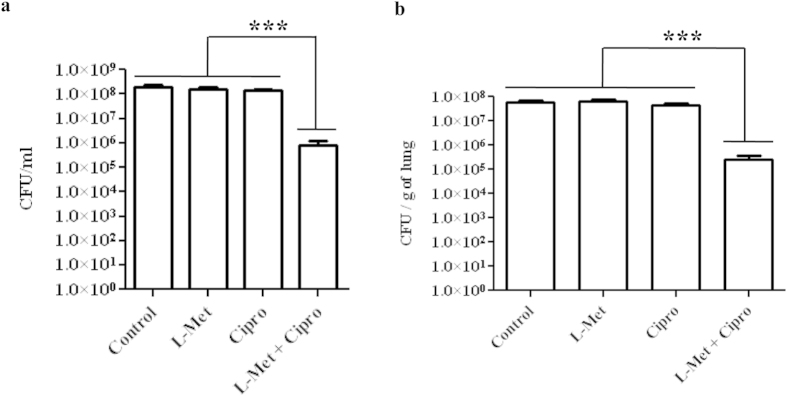
L-Met increases antibiotic sensitivity of PA biofilm *in vitro*. **(a)** 72 h old PA biofilm grown in the presence or absence of L-Met (0.5 μM) were exposed to 4 μg/ml of ciprofloxacin for 6 h. Then the cells were scraped, homogenized and plated on LB agar. The untreated biofilm samples were used as controls. **(b)** A portion of mouse lung was incubated with PA in a 24-well plate in the presence or absence of L-Met (0.5 μM) for 72 h and incubated with 4 μg/ml of ciprofloxacin for 6 h. The lungs were homogenized using bead beater, serially diluted, plated in LB agar and the number of colonies were enumerated. The Statistical significance was calculated using One-way ANOVA. Asterisks indicate statistical significance as follows: ***(p < 0.001).

Biofilms are generally associated with resistance to antibiotic treatment. We therefore examined if treatment with L-Met changed the antibiotic susceptibility of PA biofilm. L-Met treated and untreated PA biofilm were incubated with 4 μg/ml ciprofloxacin for 6 h and plated after scrapping and homogenization using a bath sonicator. The results clearly indicate that the untreated PA biofilm are resistant to ciprofloxacin whereas upon L-Met treatment they become susceptible to the same dose of ciprofloxacin (Fig. 5a). There was no difference in the total number of bacteria present in the 24 well plate due to L-Met treatment (Fig. 5a). Similarly, preformed PA biofilm in the lung also became susceptible to ciprofloxacin when a portion of a lung was incubated with PA in the presence of L-Met for 72 h and in the absence of L-Met, PA was resistant to the same concentration of ciprofloxacin (Fig. 5b). These results show that preformed biofilm are disassembled in the presence of L-Met and the free bacteria that are released from the biofilm are susceptible to the antibiotic.

### L-Met ameliorates chronic *in vivo* PA biofilm infection in combination with ciprofloxacin

**Figure 6:**
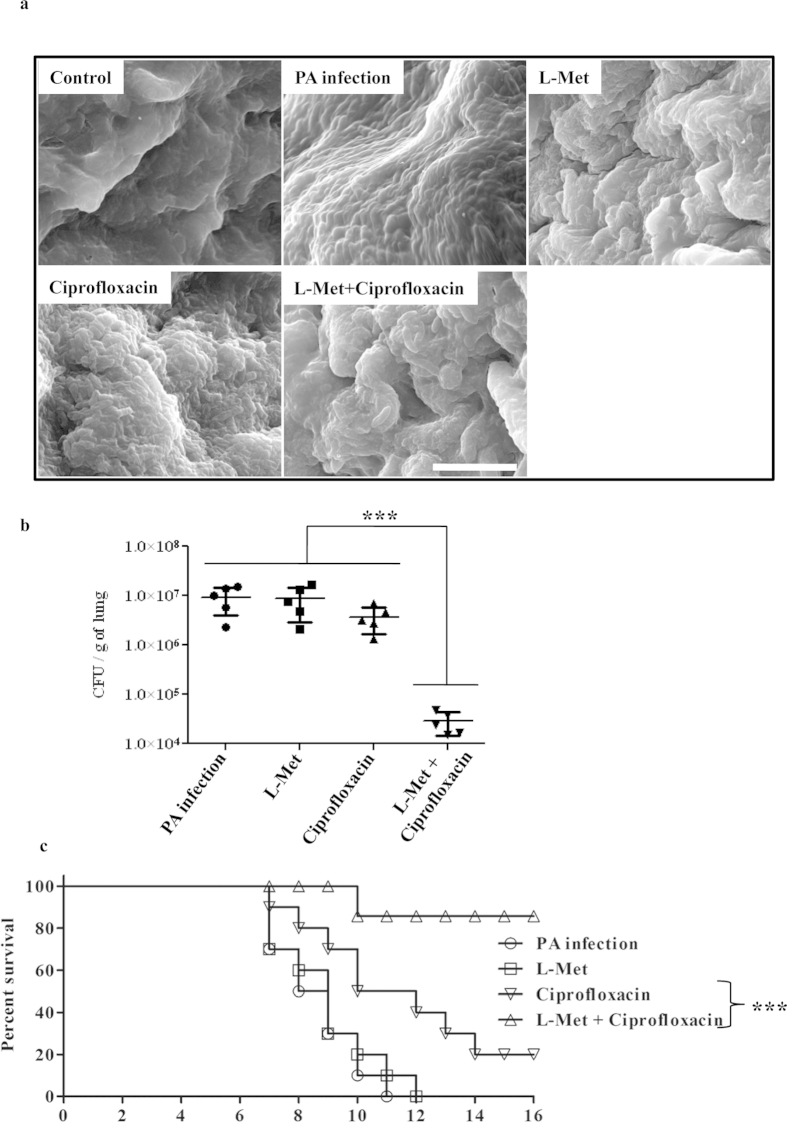
L-Methionine inhibits chronic *in vivo* PA biofilm infection in combination with ciprofloxacin. BALB/c mice were infected with PA intranasally and after3 days of infection, mice were untreated (PA infection) or treated with L-Met alone (L-Met), ciprofloxacin alone (Ciprofloxacin) or L-Met along with ciprofloxacin (L-Met + Ciprofloxacin) for further 3 days. **(a)** The mice were sacrificed and the lungs examined by SEM. Control - Uninfected mice, PA infection - Mice infected with PA and left untreated, L-Met - Mice infected with PA and treated with L-Met (5 mg/kg, intranasally). Ciprofloxacin - Mice infected with PA and treated with ciprofloxacin alone (2.5 mg/kg, via intravenous delivery), L-Met + Ciprofloxacin - Mice infected with PA and treated with L-Met and ciprofloxacin. Scale bar – 10 μm. **(b)** The number of bacteria in the lungs were determined by plating the homogenized lung tissue after 3 days of treatment. The Statistical significance was calculated using Mann-Whitney test. Asterisks indicate statistical significance as follows: ***(p < 0.001) **(c)** The survival of mice infected with PA for 3 days, then treated with ciprofloxacin with and without L-Met for further 3 days (at day 0). The animals were examined twice daily for mortality. The Statistical significance was calculated using Log-rank test. Asterisks indicate statistical significance as follows: ***(p < 0.001).

*Pseudomonas aeruginosa* (PA) chronic pneumonia infection model^33^ was used to study the effect of L-Met *in vivo*. Mice were infected with 1 × 10^7^ PA per mouse intranasally. The mice were sacrificed 3 days after the infection and the number of bacteria in the lung was calculated by plating the homogenized lung (Fig. S4a). The presence of bacteria and the formation of biofilm in lung were confirmed by SEM (Fig. S4b). After 3 days of intranasal infection, mice were treated with ciprofloxacin (2.5 mg/kg–intravenous injection) alone, L-Met alone or with L-Met + ciprofloxacin (2.5 mg/kg–intravenous injection) together for 3 days. The mice were sacrificed after 3 days of treatment, portions of the lung were visualized with SEM (Fig. 6a) and the number of bacteria in the lung was enumerated by plating (Fig. 6b). The results corroborate other studies that show that the PA infection in the lung is not sensitive to ciprofloxacin. Interestingly, the combination of L-Met and ciprofloxacin treatment could reduce the number of bacteria in the lung. Though the number of bacteria in mice treated with L-Met alone was visually lesser than the untreated mice, as observed in scanning electron micrographs (Fig. 6a), similar phenomena was not observed in the total number of bacteria in lung by CFU analysis (Fig. 6b). This may be due to the release of bacteria from the biofilm- like structure in lung due to L-Met treatment and hence the number of tissue-associated bacteria was apparently reduced in scanning electron micrographs. However, these released bacteria were not killed due to L-Met treatment and therefore there is no reduction in the total number of bacteria.

The impact of L-Met therapy on the survival of mice with PA pneumonia was also examined. At the end of the therapy (Day 0 in Fig. 6c), groups of 10 mice were examined twice daily for morbidity and mortality. Infected mice given L-Met therapy alone or left untreated died within 12 days whereas only 2 mice alone survived with ciprofloxacin alone treatment. In the group of 10 mice, 9 mice survived with ciprofloxacin + L-Met therapy. From these data, it is evident that L-Met can be used to treat PA lung biofilm infection along with antibiotics.

## Discussion

Biofilm infections are one of the life threatening conditions where treatment becomes impossible due to their resistance to antibiotic treatments. Biofilm formation occurs through different phases such as surface conditioning, attachment and colonization. Surface conditioning involves the adsorption of organic and inorganic nutrients, which facilitate bacterial attachment^34^. Initial attachment of the bacteria to the surface is reversible due to various factors such as Brownian movement and interactions between the surfaces^35^,^36^, which subsequently becomes irreversible due to the production of extracellular polymeric substances (EPS)^37^. As the bacteria grow and divide, they form primary and secondary colonies, which signal the final stage in the formation of biofilm^33^. It has been observed that biofilms are resistant to antibiotics as evidenced on urinary catheter and urethral stent surfaces *in vitro* and *in vivo*^38^,^39^,^40^.

D-amino acids are known to disassemble the established biofilms^23^. When we investigated the effect of different L-/D- amino acids on *Pseudomonas aeruginosa* biofilm, consistently we observed that the L-Methionine treated biofilm culture was less viscous compared to the other cultures at 72 h. This lead to the hypothesis that L-Met has an effect on extracellular DNA (eDNA). It is already known that in PA, extracellular DNA is required for the biofilm formation^8^,^10^ and external DNase treatment suppress the biofilm formation^8^,^41^. PA biofilm formation was diminished at 72 h when treated with 0.5 μM L-Met. Previously it was reported that L-/D-Methionine did not inhibit PA biofilm formation^25^. Nevertheless, in that report^25^, the authors have tested 0.5 mM to 10 mM concentrations of amino acids to assess the effect. In our study, we observed that only at 0.5 μM concentration of L-Met, all the phenotypes were observed. Quorum sensing genes are known to play a major role in the establishment of biofilm and eDNA^42^. Since quorum sensing is highly sensitive to population density and signaling molecules, it is possible that the effect of L-Met might be mediated by quorum sensing-associated pathways.

Interestingly, when the plasmid (pFPV/mcherry)/U-Sup were incubated with the L-Met-Sup, a smear was observed and there was a slight increase in the mobility. This indicated the presence of DNase activity in the L-Met-Sup. QPCR results confirmed the up-regulation of four *DNase* genes at mRNA level. Hence, the DNase are not only transcriptionally up-regulated but are active enough to degrade the eDNA in the outside environment. The presence of a smear in the L-Met-Sup (Fig. 3a,b) confirms that the eDNA is degraded by secreted DNase. Addition of EDTA arrests DNase activity in the culture supernatant. It is known that, *Bacillus licheniformis* uses secreted nucleases as a strategy to disperse established biofilms and to prevent biofilm formation of competitors^43^. DNase treatments have been exploited to inhibit the biofilm formation and disassemble the established biofilms^44^,^45^. Here, we have observed concentration dependent phenomenon of L-Met to disrupt PA biofilm. Concentration dependent differential activity was observed for different molecules in bacterial and mammalian systems^46^,^47^,^48^. We have observed the disruption of PA biofilm at specific low concentration of L-Met. Since L-Met is an amino acid and it can be utilized by bacteria as a nutrient source, we hypothesize that, at higher concentration L-Met is not providing the specific cue to induce DNase whereas, at low concentrations bacteria cannot detect L-Met as a signal for the cue. However, the mechanism of inducing DNase by L-Met is not yet understood.

We found that four different *DNase* genes were highly up-regulated. Though DNase activity was observed and confirmed in the culture supernatant, the mechanism of DNase secretion is not known. There are 3 different possibilities of DNase activity in the culture supernatant. In our first hypothesis, DNase may be secreted by unknown mechanism using the secretion system present in PA. Only EddB, which plays an important role in eDNA degradation, was predicted as a secretory protein using bioinformatics tool. The other DNases are predicted as cytoplasmic and their functions with respect to eDNA are not studied. In the second hypothesis, DNase is up-regulated in protein level inside the bacteria and upon autolysis or other lysis mechanism of PA, DNase are released into the culture media and show their activity. It is also possible that L-Met increase activity of DNases released from lysed bacteria. We have tested this hypothesis by incubating the L-Met treated and untreated culture supernatant with L-Met and we did not find any significant difference in the DNase activity (Fig. S5). Since we observe DNase activity only after 48 h, the second hypothesis would be more appropriate. However, the mechanism needs to be addressed.

We have used L-Met as a therapeutic strategy to disrupt biofilm *in vitro* and *in vivo*. When the PA biofilm was treated with ciprofloxacin, there was no reduction in the number of bacteria, whereas ciprofloxacin treatment along with L-Met could significantly reduce the number of bacteria in all the cases. Further experiments were carried out to check the effect of L-Met in biological biofilm infection. Bacterial biofilm infections are commonly observed in cystic fibrosis patients and the most common causative organisms for infections in cystic fibrosis are *P.aeruginosa*, *S.aureus* and *H.influenza*^49^. The main reason for the persistence of infection in cystic fibrosis is the ability of PA to form and reside within recalcitrant biofilm^50^. We have infected mice with PA through the nasal route and established the biofilm model in the lung. When the mice were treated with only ciprofloxacin there was no decrease in the PA burden, but in combination with L-Met treatment, the infection was reduced.

Our study reveals for the first time that L-Met induces DNase synthesis in PA and hence the biofilm formation is hindered. For cystic fibrosis (CF), DNaseI treatment is already established^51^ and other research is being carried out to enhance the efficiency of this treatment in CF patients. It has been shown that in combination with poly-aspartic acid or gelsolin, the DNase1 activity is increased in the CF sputum^52^. DNase1 functionalized nanoparticle loaded with antibiotic showed enhanced efficiency on killing of PA in CF sputum^53^. Our findings can be extended to study the effect of L-Met on PA infection in CF condition. Our data clearly demonstrates that L-Met can be used as an adjunct therapy to treat PA biofilm infection *in vivo* in combination with antibiotic treatments.

## Conclusion

In the present study, we found for the first time that L-Methionine at low concentrations (~0.5 μM) and not at higher concentrations inhibited the *Pseudomonas aeruginosa* biofilm formation. We also found that L-Met could disassemble the established 48 h and 72 h old PA biofilms. L-Met induced DNase secretion may be the possible mechanism for the biofilm inhibition and disassembly. Importantly, we have shown that intranasal L-Met treatment along with antibiotics can cure chronic PA lung infection in mice. Hence, the nasal spray or drop formulations with L-Met along with antibiotic treatment can open up better avenues to treat CF cases or PA induced lung infections.

## Methods

### *Pseudomonas aeruginosa* (PA) biofilm formation

*Pseudomonas aeruginosa* strain PA01^54^ was grown in Luria broth at 37 °C in a shaker at 180 rpm and subsequently maintained on Luria agar. For PA biofilm formation, 1 × 10^4^ stationary phase cells were inoculated into 2 ml of Luria broth without NaCl and kept at 25 °C in static condition in a 24-well plate in the presence or absence of different concentrations of L-Methionine for different duration.

### Crystal violet (CV) staining of biofilms

24-well plate containing biofilm on the inner walls and the bottom of the plate was confirmed by CV staining^55^. After different time points of incubation at static condition, the culture was removed and the wells were washed with phosphate buffered saline (PBS) three times to remove the planktonic cells. The wells were dried and stained with 1% (w/v) crystal violet for 15 minutes at room temperature. After three PBS washes, the bound crystal violet was solubilized with 200 μl of absolute ethanol. The optical density was determined at 545 nm (OD_545_).

### Growth curve

1 × 10^4^ stationary phase PA were inoculated into 10 ml of Luria broth without NaCl or M9 minimal media in the presence (0.5 μM) or absence of L-Met and kept at 37 °C in shaking condition (180 rpm) and at different time points optical density was determined at 600 nm (OD_600_) (SpectraMax 340PC, Molecular Devices).

### Scanning electron microscopy (SEM)

A sterile cover slip was placed in a 24-well plate and the PA biofilm was formed as described before. Cover slips with biofilm were fixed with 2.5% (v/v) glutaraldehyde and the samples were dehydrated with increasing concentrations of ethanol for 2 min each. The samples were stored in vacuum until use. Prior to analysis by Field emission SEM (FEI-SIRION, Eindhoven, Netherlands), the samples were subjected to gold sputtering (JEOL JFC 1100E Ion sputtering device).

### Disassembly of established PA biofilm

Different concentrations of L-Met (0.05 μM, 0.5 μM, 5 μM & 50 μM) was added to 48 h and 72 h old biofilm in a 24-well plate and incubated for 24 h at 25 °C static condition. CV staining was performed as described before to measure the biomass.

### Agarose gel electrophoresis

Agarose gel (1%) electrophoresis was performed to detect the presence of extracellular DNA (eDNA) in the biofilm culture media. 72 h old PA biofilm was established in a 24-well plate as described before. The total number of bacteria (suspension form in the media + adherent biofilm form) in the plate was determined by sonication and plating. The media in the 24-well plate was normalized to total number of bacteria, centrifuged at 2000 g for 5 min and the supernatant [Untreated culture (U-Sup) & L-Met treated culture (L-Met-Sup)] was loaded in the agarose gel and visualized. U-Sup/L-Met-Sup was treated with L-Met (0.5 μM final concentration), DNase and RNase (Fermentas) for 1 h at 37 °C and then loaded in the agarose gel for visualization.

### Measurements of extracellular DNA (eDNA)

eDNA in the PA culture was determined as described elsewhere^26^. Briefly, treated or untreated PA culture media were centrifuged at 12000 g for 5 min and the supernatant was transferred to a new microfuge tube. At a final concentration of 250 mM NaCl in the sample, the eDNA was precipitated by adding ethanol in 2:1 ratio and resuspended in 100 μl of TE buffer. The concentration of eDNA was quantified by dsDNA quantification kit as per the manufacturer’s protocol (Quant-iT™, PicoGreen dsDNA Assay Kit, Life technologies). Briefly, 100 μl of diluted eDNA was mixed with 100 μl of PicoGreen reagent and incubated for 5 min at 25 °C before measuring the fluorescence intensity with 480 nm excitation and 520 nm emission (TEKAN, Infinite M200 Pro, Switzerland). The eDNA concentration (μg/ml) was quantified by comparing with the standard and the measured amount of eDNA was plotted as μg/ml.

### Viscosity measurements

Viscosities (η) of all samples were determined at 20 °C using a Discovery Hybrid Rheometer-3 (DHR-3) (TA instruments, New Castle DE, USA) as per the manufacturers’ protocol. Measurements were taken using an anodized aluminum cone (4 mm diameter, 2 degree).

### DNase activity

100 μl of 72 h old PA culture supernatant (U-Sup or L-Met-Sup) were incubated with equal volume of pFPV/mcherry (1 μg/μl) with or without 0.1% EDTA for 1 h at 37 °C. The samples were loaded in 1% agarose gel and visualized. In the other experiment, 100 μl of U-Sup and L-Met-Sup were mixed and incubated for 1 h at 37 °C, loaded in 1% agarose gel and visualized.

### QPCR for *DNase* genes expression

Assessment of the expression of different *DNase* genes was carried out using QPCR. Briefly, RNA was isolated from 72 h old PA biofilm culture grown at 25 °C static condition with/without L-Methionine using TRIzol (Life Technologies) as per manufacturer’s protocol. The isolated RNA was reverse transcribed using random hexamers (NEB) and Tetro reverse transcriptase (Bioline) as per standard protocol. The cDNA was diluted and analyzed for the presence different *DNase* genes using specific primers (Table 1) by QPCR SYBR® FAST qPCR Master Mix (Kapa Biosystems) in an Applied Biosystems® ViiA™ 7 Real time PCR instrument. Expression was normalized to the housekeeping gene *16s rRNA.*

### Motility assays

#### Swarming motility

The swarming motility assay was performed on a Petri dish as described elsewhere^32^. Briefly, stationary cultures of PA were washed twice with PBS and the OD was adjusted to 3.0. 5.0 μl of the culture was carefully spotted onto the centre of agar plates [M9 minimal plates (0.2% Glucose, 0.5% CAA, and 0.5% agar)] containing various concentrations of L-Methionine (0.05, 0.5, 5 & 50 μM). The plates were incubated at 30 °C for 24 h, and the colony diameter was measured and plotted. In another experiment, 5 μl of 72 h old PA biofilm culture grown in LB, LB+L-Met (0.5 μM), LB + EDTA (0.1%) or LB+L-Met+EDTA were inoculated in the centre of M9 minimal plates with or without L-Met (0.5 μM). After 24 h of incubation at 30 °C, the colony diameter was measured and plotted.

#### Twitching motility

Twitching motility assay was performed as described elsewhere^56^. Briefly, PA strain was stab inoculated through a 1% LB agar plate with or without L-Met (0.5 μM) and incubated for 12 h at 37°C. The plate was flooded with 20% methanol and 10% acetic acid solution to visualize the zone of interstitial biofilm expansion at the agar and Petri dish interface. The surface area calculated by measuring the longest (d_l_) and shortest (d_s_) diameters of each interstitial biofilm and using the following equation:


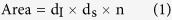


### *In vitro* antibiotic sensitivity assay

PA biofilm after 72 h of incubation with or without L-Met (0.5 μM) in a 24-well plate were exposed to 4 μg/ml of ciprofloxacin under shaking conditions at 180 rpm, 37 °C for 6 h. Then the cells were scraped, homogenized by sonication for 10 minutes in a bath sonicator and plated on LB agar^57^. The untreated biofilm samples were used as controls. Similarly, a portion of mouse lung was incubated with PA in a 24-well plate in the presence or absence of L-Met (0.5 μM) for 72 h. The lungs were washed thrice with PBS and incubated with 4 μg/ml of ciprofloxacin under shaking conditions at 180 rpm, 37 °C for 6 h. The lungs were homogenized using bead beater, serially diluted, plated in LB agar and the number of colonies were enumerated.

### *In vivo* experiments

#### Animal ethics

All the experiments were carried out in accordance with the approved guidelines of institutional animal ethics committee at Indian Institute of Science, Bangalore, India (Registration No: 48/1999/CPCSEA). All procedures with animals were carried out in accordance with the institutional rules for animal experimentation.

#### Chronic PA lung infection mouse model

BALB/c mice bred and housed at the Central Animal Facility of Indian Institute of Science (IISc) were used for all the *in vivo* experiments. The mice used for the experiments were 6–8 weeks old. All procedures with animals were carried out in accordance with the Institutional rules for animal experimentation. All animal experiments were approved by the Institutional Animal Ethics Committee and the National Animal Care Guidelines were strictly followed. Agarose bead infection method was used to establish chronic pulmonary infection in mice as described elsewhere^33^. Briefly, adult BALB/c mice were infected by intranasal injection of bacterial beads prepared by mixing 1 ml of overnight culture of PA with 9 ml of PBS and 25 ml of 2% (w/v) agarose. The mixture was stirred for 30 min after adding 500 ml of mineral oil at 50 °C and the mixure was cooled down to 25 °C. The agarose beads were washed once with 0.5% deoxycholic acid, sodium salt (SDS) in PBS, once with 0.25% SDS, and four times with PBS. The number of viable bacteria was enumerated and the beads were stored for not more than 48 h at 4 °C.

BALB/c mice were intranasally infected with 50 μl of 1:10 dilution bead slurry. On day 3 post infection, different cohorts of mice were treated with ciprofloxacin alone (2.5 mg/kg – intravenous injection – once per day), L-Met treatment alone (5 mg/kg - intranasal - once per day), or L-Met (5 mg/kg - intranasal - once per day) and ciprofloxacin treatment (2.5 mg/kg – intravenous injection – once per day) for 3 days. The mice were sacrificed after 3 days, and the lungs was aseptically removed, weighed and homogenized in sterile PBS. The homogenate was plated using serial dilutions onto LB agar to determine the bacterial load. A small portion of the lung was fixed with 2.5% (v/v) glutaraldehyde and processed with increasing concentrations of alcohol (10, 20, 30, 50, 70, 80, 90 and 100%) for 2 min and analysed with SEM as described. For survival assay, same treatment was given (n = 10 mice/group) and the mice were monitored twice daily for 15 days for morbidity and mortality.

## Additional Information

**How to cite this article**: Gnanadhas, DP *et al.* Chronic lung infection by *Pseudomonas aeruginosa* biofilm is cured by L-Methionine in combination with antibiotic therapy. *Sci. Rep.* **5**, 16043; doi: 10.1038/srep16043 (2015).

## Supplementary Material

Supplementary Information

## Figures and Tables

**Table 1:** *DNase* specific primers used for QPCR analysis.

*DNase* Genes	Primers	
xthA	Fwd	5′ CGAATGGCTGGCTACCTTGA 3′
Exodeoxyribonuclease III	Rw	5′ CGGTAGTCGAACCAGCTGAA 3′
endA	Fwd	5′ GTTTGTAGGCCTTTTCGCCC 3′
DNA-specific endonuclease I	Rw	5′ CGTAGAGCTTCCAGCCGATT 3′
nth	Fwd	5′ GTCGGGGTGAACAAGGCTAC 3′
Endonuclease III	Rw	5′ TCGCCTTGCTGTTGTAGAGG 3′
recJ	Fwd	5′ ACTTTCCCGAGCCGATGTTC 3′′
Single-stranded-DNA-specific exonucleaseRecJ	Rw	5′ GCATTCGCTTTTCAGCACCA 3′
sbcD	Fwd	5′ AAGACTCCGAGCGAAACCTG 3′
ExonucleaseSbcD	Rw	5′ CGACTTTCTGTGGCTTGTGC 3′
sbcB	Fwd	5′ AAGCAGATCCAGGTCAACCG 3′
Exodeoxyribonuclease I	Rw	5′ AACAGCTCGGCTTTTTGCTG 3′
eddB	Fwd	5′ CCAGCTTCAACGTGCTCAAC 3′
Extracelullar DNA degradation protein, EddB	Rw	5′ TTCTGCCGTTGGAACTCCTC 3′

**Table 2:** Different DNase in *Pseudomonas aeruginosa.*

DNase	Size	Name	Predicted Cellular Localization using ProteinPredict
SbcB	476 aa	Exonuclease I	Cytoplasmic
EndA	237 aa	DNA-specific endonuclease I	Cytoplasmic
EddB	779 aa	Extracelullar DNA degradation protein	Secreted
RecJ	571 aa	Single-stranded-DNA-specific exonuclease	Cytoplasmic
XthA	270 aa	Exonuclease III	Cytoplasmic
Nth	212 aa	Endonuclease III	Cytoplasmic
SbcD	409 aa	Exonuclease	Cytoplasmic
