# Supplementary material for: Chronic lung infection by Pseudomonas aeruginosa biofilm is cured by L-Methionine in combination with antibiotic therapy
Source: Sci Rep. 2015 Nov 2;5:16043. doi: 10.1038/srep16043 (PMC4629202; doi:10.1038/srep16043)
Supplement: Supplementary Information [file srep16043-s1.pdf]

SUPPORTING INFORMATION

Title: Chronic lung infection by Pseudomonas aeruginosa biofilm is cured by L-Methionine in combination with antibiotic therapy

Divya Prakash Gnanadhas[1,2,†*], Monalisha Elango[1*], Akshay Datey[1,2,3], Dipshikha Chakravortty[1,#]

[1]Department of Microbiology and Cell Biology, Indian Institute of Science, Bangalore, India.

[2]Department of Aerospace Engineering, Indian Institute of Science, Bangalore, India.

*Equal contribution

[3]The Bioengineering Program, Indian Institute of Science, Bangalore, India.

# Corresponding author

Dipshikha Chakravortty

E.mail: dipa@mcbl.iisc.ernet.in,

Telephone: +91 80 22932842, Fax: +91 80 23602697

[†] Present Address: Department of Pharmacology and Experimental Neuroscience, University of Nebraska Medical Center, Omaha, NE, USA.

Figure S1

a

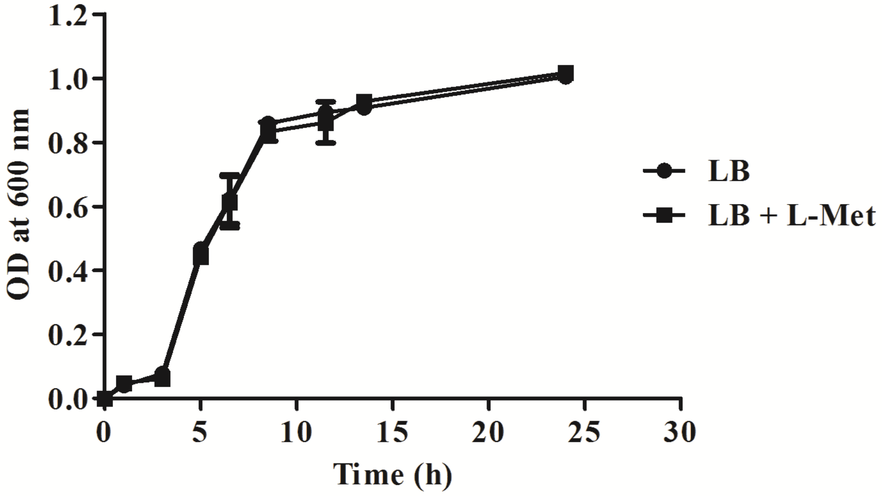

b

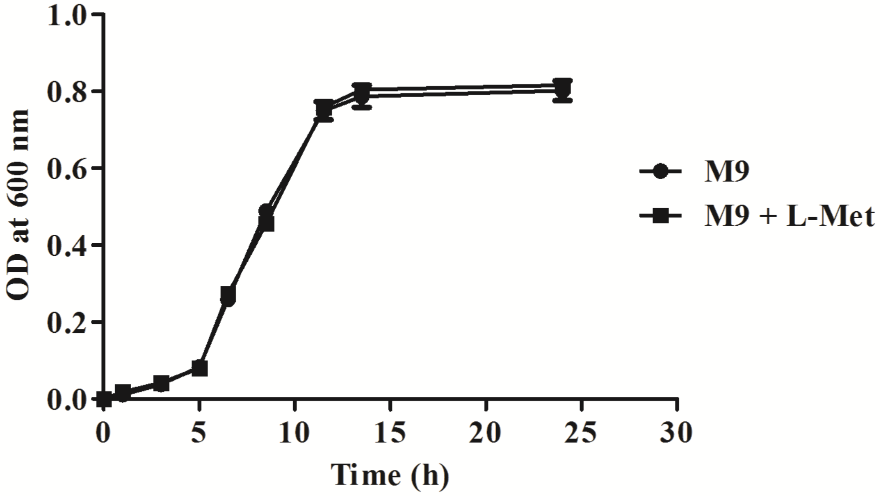

Figure S1. Growth cure. 1×10^4 stationary phase PA were inoculated into 10 ml of **(a)** Luria broth without NaCl **(b)** M9 minimal media in the presence or absence of 0.5 μM L-Met and kept at 37°C in shaking condition (180 rpm) and at different time points optical density was determined at 600 nm.

Figure S2

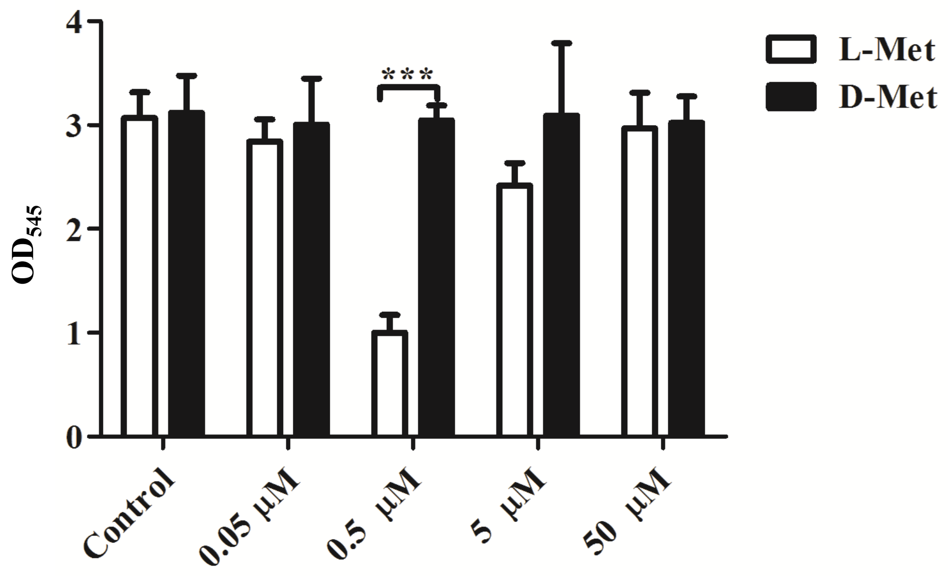

Figure S2. Effect of D-Met on PA biofilm. PA was incubated with different concentrations of L-Met or D-Met and at 72 h, the biomass was determined by crystal violet staining. . The Statistical significance was calculated using One-way ANOVA. Asterisks indicate statistical significance as follows: *** ($p<0.001$)

Figure S3

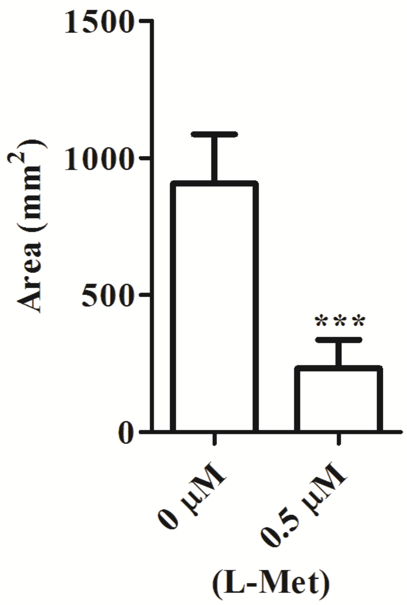

Figure S3. L-Met inhibits *Pseudomonas aeruginosa* twitching motility. Stationary PA was spotted on 1% LB agar plates with or without 0.5 μM L-Met and the plates were incubated at 37°C for 24 h. Areas of interstitial biofilms were measured and presented as the mean±SD from three independent experiments performed in triplicate. The Statistical significance was calculated using students' t test. Asterisks indicate statistical significance as follows: *** (p<0.001)

Figure S4

a

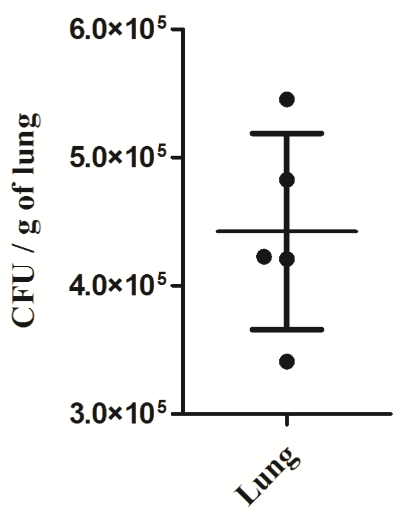

b

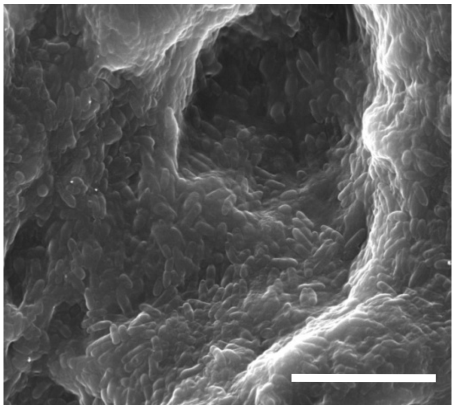

Figure S4. Establishment of PA lung infection model. BALB/c mice were infected with PA via the intranasal route for 3 days. **(a)** The mice were sacrificed and the lungs were removed, homogenized, and plated onto LB agar. **(b)** SEM images of infected lung containing PA biofilm. Scale bar: 10 μm.

Figure S5

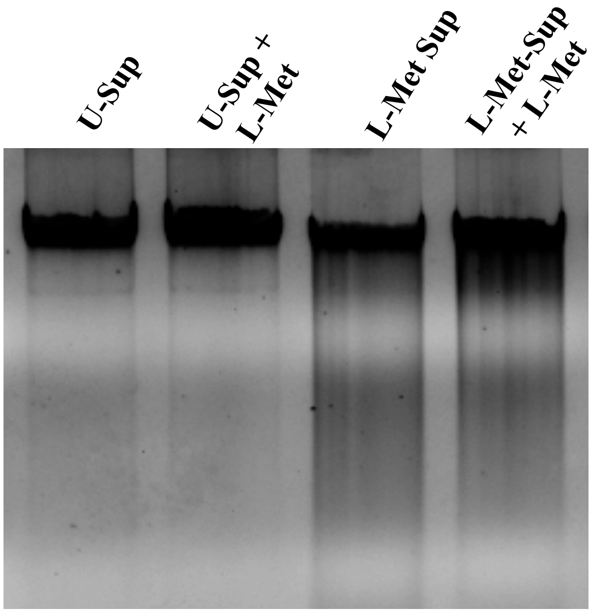

Figure S5. Effect of L-met on DNase activity. 100 μl of 72 h old U-Sup or L-Met-Sup were incubated with L-Met (0.5 μM) for 1h at 37°C and the samples were loaded in 1% agarose gel and visualized.
